# Obesity enhances the response to neoadjuvant anti‐PD1 therapy in oral tongue squamous cell carcinoma

**DOI:** 10.1002/cam4.7346

**Published:** 2024-06-24

**Authors:** Xiyan Tan, Guoli Li, Honghao Deng, Guoming Xiao, Yaqin Wang, Chenzhi Zhang, Yanfeng Chen

**Affiliations:** ^1^ State Key Laboratory of Oncology in South China, Guangdong Provincial Clinical Research Center for Cancer Sun Yat‐sen University Cancer Center Guangzhou P.R. China; ^2^ Department of Head and Neck Surgery Sun Yat‐sen University Cancer Center Guangzhou P.R. China; ^3^ Department of Radiation Oncology Sun Yat‐sen University Cancer Center Guangzhou P.R. China; ^4^ Department of Colorectal Surgery Sun Yat‐sen University Cancer Center Guangzhou P.R. China

**Keywords:** fatty acid metabolism, immunotherapy, obesity, oral tongue squamous cell carcinoma, prognosis

## Abstract

**Objectives:**

Previous studies have demonstrated that obesity may impact the efficacy of anti‐PD1 therapy, but the underlying mechanism remains unclear. In this study, our objective was to determine the prognostic value of obesity in patients with oral tongue squamous cell carcinoma (OTSCC) treated with pembrolizumab and establish a subtype based on fatty acid metabolism‐related genes (FAMRGs) for immunotherapy.

**Materials and Methods:**

We enrolled a total of 56 patients with OTSCC who underwent neoadjuvant anti‐PD1 therapy. Univariate and multivariate Cox regression analyses, Kaplan–Meier survival analysis, and immunohistochemistry staining were performed. Additionally, we acquired the gene expression profiles of pan‐cancer samples and conducted GSEA and KEGG pathway analysis. Moreover, data from TCGA, MSigDB, UALCAN, GEPIA and TIMER were utilized to construct the FAMRGs subtype.

**Results:**

Our findings indicate that high Body Mass Index (BMI) was significantly associated with improved PFS (HR = 0.015; 95% CI, 0.001 to 0.477; *p* = 0.015), potentially attributed to increased infiltration of PD1 + T cells. A total of 91 differentially expressed FAMRGs were identified between the response and non‐response groups in pan‐cancer patients treated with immunotherapy. Of these, 6 hub FAMRGs (ACSL5, PLA2G2D, PROCA1, IL4I1, UBE2L6 and PSME1) were found to affect PD‐1 expression and T cell infiltration in HNSCC, which may impact the efficacy of anti‐PD1 therapy.

**Conclusion:**

This study demonstrates that obesity serves as a robust prognostic predictor for patients with OTSCC undergoing neoadjuvant anti‐PD1 therapy. Furthermore, the expression of 6 hub FAMRGs (ACSL5, PLA2G2D, PROCA1, IL4I1, UBE2L6 and PSME1) plays a pivotal role in the context of anti‐PD1 therapy and deserves further investigation.

## 1 INTRODUCTION

The most common head and neck malignancies are thought to originate in the mucosal epithelium of the oral cavity, pharynx, and larynx.^1^ According to the statistical study, tongue cancer in the United States in 2024 contains 19,360 newly diagnosed cases and 3320 deaths.^2^ One of the most prevalent tongue malignancies is oral tongue squamous cell carcinoma (OTSCC), and in recent years, its frequency has increased.^3^, ^4^

Presently, OTSCC is treated with surgery and chemotherapy, radiotherapy, immunotherapy and combination treatment.^4^, ^5^ Recently, there have been significant advancements in the development of drugs that target the interaction between the receptor known as programmed death‐1 (PD‐1) and its ligands, namely programmed death‐ligand 1/2 (PD‐L1/L2). These drugs have demonstrated clinical effectiveness across a wide range of tumor types.^6^, ^7^, ^8^ To date, the utilization of anti‐PD1 therapy has exhibited promising outcomes in the treatment of HNSCC patients experiencing tumor progression.^9^ In recent years, inhibitors of PD‐1, are approved for use in patients with progressive HNSCC, these drugs have a response rate of about 20% and an overall survival benefit compared with chemotherapy.^10^, ^11^, ^12^ Afterward, several clinical randomized studies proved that pembrolizumab prolong the overall survival of patients with progressive HNSCC.^12^, ^13^ Additionally, anti‐PD1 therapy was used in the neoadjuvant therapy setting in untreated patients with advanced tumors with promising results.^6^, ^14^, ^15^ Moreover, neoadjuvant anti‐PD1 therapy holds potential in achieving tumor reduction while preserving organ function and facial appearance, thereby maximizing patients benefits, which enable more advanced patients with opportunities for surgical treatment. In spite of the advancements made in research and treatment over the past 10 years, The low response rate among patients with HNSCC poses a significant limitation to the effectiveness of immune checkpoint inhibitors (ICIs) treatment. Additionally, both clinics and biomedical science face considerable challenges in dealing with OTSCC.^16^, ^17^ Hence, it is crucial to promptly discover potential biomarkers capable of precisely predicting prognosis and forecasting the effectiveness of immunotherapy.

In the past few years, incidence of obesity has increased significantly, and population data link obesity to the increased incidence of several common cancers.^18^, ^19^ Thus, obesity emerge as a pressing global concern. Previous study suggested that obesity associated with many of diseases has been linked to dysfunction of the immune system.^20^ Moreover, T lymphocytes play a crucial role in the immune system, regulating key elements of an immune response.^21^ In addition, checkpoint blockade therapies aimed at T cell responses are proving to be effective in the treatment of cancer patients in the clinic.^22^ As it is known to all, obesity can lead to chronic inflammation, which promote an exhausted T cell phenotype.^23^ Similarly, the obese state with chronic inflammation and subsequent generation of exhausted T cells may enhance tumor progression while concurrently promoting an environment conducive to ICIs.^24^ Preclinical studies demonstrated that obesity enhanced tumor growth, which was associated with dysfunctional CD8 T cells.^25^, ^26^ However, in several tumor models treatment of obese mice with anti‐PD1 slowed down tumor growth or led to complete tumor resistance.^27^, ^28^ The first major study to report this finding with ICB was that obesity was found to be associated with a significant reduction in disease progression and death risk in patients with metastatic melanoma who received immune checkpoint blockade (ICB) treatment.^29^ A recent study also showed that obese Asian patients with advanced non‐small cell lung cancer who received immune checkpoint inhibitors had better overall survival independent of muscle or fat mass.^30^ Therefore, obesity has a significant impact and checkpoint blockade therapy has the potential to cure cancer, to enhancing the utilization of these therapies in the growing number of obese patients, it is imperative to acquire a more comprehensive understanding regarding the impact of obesity on T cells.

Obesity could also alter tumor lipid metabolism.^31^ As a crucial intermediate product in lipid metabolism, fatty acid metabolism (FAM) plays an indispensable role in numerous biological activities and holds great potential as an immunotherapy target.^32^, ^33^ And previous studies have identified a potential correlation between fatty acid metabolism and the effectiveness of immunotherapy as well as prognosis in patients with malignancies in.^34^ For example, fatty acid metabolism‐related genes (FAMRGs) are potentially useful for predicting prognosis and immunotherapy response in bladder cancer.^35^ The latest research demonstrates that the reprogramming of fatty acid metabolism has a significant impact on the phenotype of immune cells infiltrating the microenvironment of melanoma. Furthermore, the identification of biomarkers for molecular subtypes in FAM can independently predict prognosis and immunotherapy response in melanoma patients.^36^, ^37^ However, the prognostic and therapeutic effect of abnormal lipid metabolism throughout the body and FAM‐related biomarkers in OTSCC remains unexplored.

**FIGURE 1:**
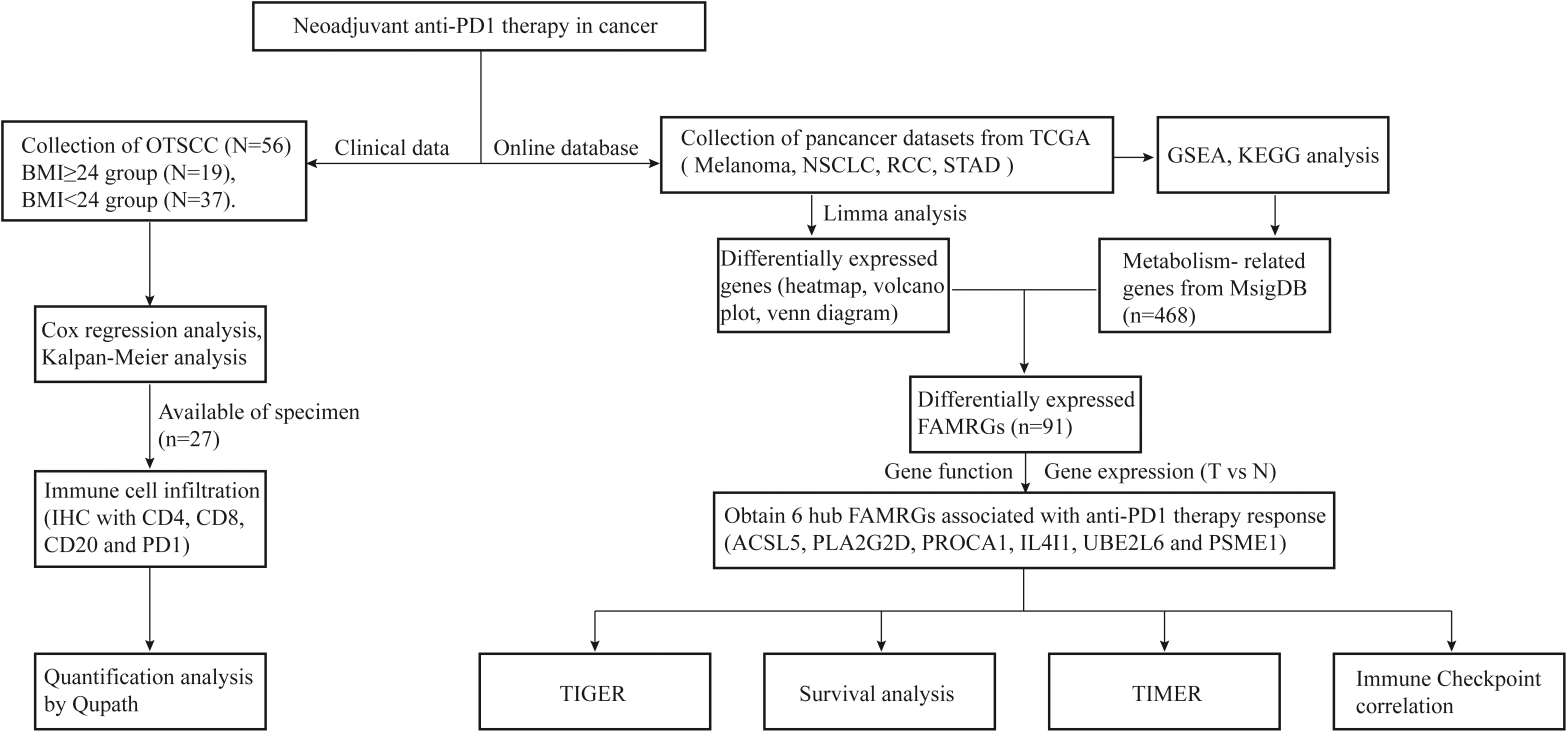
The workflow of the current work.

Our study found that obese patients have more PD1+ T cell infiltration, so they could benefit more from immunotherapy. We also identified that 6 FAMRGs were positively associated with the efficacy of immunotherapy, which may be used as molecular indicators to predict the efficacy of ICI. Our analysis process was shown in Figure 1.

## 2 MATERIALS AND METHODS

### 2.1 Clinical data collection

With the approval of the Institutional Review Board of Sun Yat‐sen University Cancer Center (approved number: B2022‐221‐Y01, approved data: 2023‐6‐15), this study was granted a waiver of informed consent. The data of 56 OTSCC patients receiving anti‐PD1 therapy from October 2013 to April 2022 were retrospectively reviewed in Sun Yat‐sen University Cancer Center.

The stringent eligibility criteria were as follows: the resected specimen demonstrated the presence of histologically confirmed squamous cell carcinoma; the primary lesion had to be situated in the tongue; pathologic stages cII, cIII, cIVA, cIVB (NCCN Guidelines 1.2022 edition); sufficient organ functionality; absence of clinically significant abnormal findings on electrocardiography. Patients less than 5 months follow‐up were excluded. The initial measurements of BMI, OS and PFS were obtained for the 56 patients. PFS was determined as the duration between the initiation of anti‐PD1 therapy and either the occurrence of disease progression on radiological imaging or death due to disease. OS was determined as the period from the initiation of anti‐PD1 therapy until death resulting from the disease or until it censored at the last follow‐up.

### 2.2 Gene set enrichment analysis (GSEA) and Kyoto Encyclopedia of Genes and Genomes (KEGG) analyses

We obtained RNA sequence and corresponding clinical data of patients received PD‐1 inhibitors treatment from the The Cancer Genome Atlas database (TCGA, https://portal.gdc.cancer.gov/) and NCBI‐GEO DataSets (https://www.ncbi.nlm.nih.gov), including 49 response samples and 42 non‐response samples in melanoma, 8 response samples and 19 non‐response samples in non‐small cell lung cancer (NSLCL), 4 response samples and 7 non‐response samples in renal cell carcinoma (RCC), and 21 response samples and 57 non‐response samples in stomach (STAD) samples.

GSEA was conducted to explore the distinct pathways associated with the differential gene expression in cancer patients. The GSEA analysis was conducted with cohort PRJEB23709, GSE135222, GSE67501, PRJEB25780 in melanoma, NSCLC, RCC and STAD, respectively. The selection of HALLMARK gene sets and KEGG gene sets was based on statistical significance, as indicated by the normalized enrichment score (NES), false discovery rate (FDR), and *p*‐value. Statistical significance was considered when FDR ≤0.25.

The analysis was conducted using limma package, wherein differentially expressed genes (DEGs) were identified by applying a significance threshold of *p*‐value <0.05 and |log2 FC > 1 to distinguish between response and non‐response samples. Subsequently, the biological functions associated with these DEGs in pan‐cancer were systematically investigated using DAVID (https://david.ncifcrf.gov), a tool available for KEGG pathway analyses. *p*‐value was considered statistically significant.

### 2.3 GEPIA

The GEPIA database (http://gepia.cancer‐pku.cn/) a wealth of RNA sequencing expression data collected from 9736 tumors and 8587 normal samples sourced from the TCGA and GTEx databases. In recent study, we set out to examine the association between the expression of FAMRGs and PD‐1 levels by utilizing the GEPIA database to calculate Spearman's correlation coefficients.

### 2.4 Analysis of fatty acid metabolism‐associated genes in TIMER

The TIMER database (https://cistrome.shinyapps.io/timer/),^38^, ^39^ a comprehensive tool for analyzing tumor‐infiltrating immune cells, was employed to examine the correlation between the expression levels of FAMRGs and both tumor purity and tumor‐infiltrating immune cells in HNSCC.

### 2.5 Data processing of FAMRGs

We obtained the GMT file containing the gene set associated with the metabolic process of fatty acids from the Molecular Signatures Database (MsigDB) (https://www.gsea‐msigdb.org/gsea/msigdb/genesets.jsp) database in format of a GMT file, and 468 fatty acid metabolism‐related genes (FAMRGs) was found (Table S1).^40^ Then, differentially expressed genes (DEGs) of 4 cancer database between anti‐PD1 therapy responder specimen and non‐responder specimen were identified via limma analysis online tool (http://sangerbox.com/home.html) with |log2 FC|>1 and *p*‐value <0.05. Finally, we used Venn software online (http://bioinformatics.psb.ugent.be/webtools/Venn/) to identify the fatty acid metabolism‐related genes (FAMRGs) in the DEGs of 4 cancer datasets.

### 2.6 Immunohistochemistry staining

The tissue specimens were preserved in a 10% solution of formalin and then embedded in paraffin wax. Subsequently, sections measuring 5 μm were obtained from the tissue blocks. These sections underwent deparaffinization using xylene, and dehydration though a sequence of alcohol concentrations (75%, 85%, 95%, 100%). To retrieve antigens, EDTA was employed, followed by blocking with goat serum at a concentration of 5%. The tissue sections were subsequently subjected to incubation with primary antibodies targeting CD4 (ZA‐0519, ZSGB‐BIO, China), CD8 (ZA‐0508, ZSGB‐BIO, China), CD20 (ZM‐0039, ZSGB‐BIO, China), PD‐1 (ZM‐0381, ZSGB‐BIO, China). Then, the tissue sections were subjected to a 2‐h incubation at room temperature with secondary antibodies (PV‐6000, ZSGB‐BIO, China). Following the incubation, the tissue sections with DAB. After the applications of a staining process, the sections underwent digital scanning using a scanner from Leica Biosystems made in Germany. Subsequently, analysis was conducted utilizing a workstation named Qupath, employing nuclear and membranal algorithms trained by pathologists. The protein expression was assessed based on the density of positive immune cells per square millimeter using Qupath image analysis.

### 2.7 Statistical analysis

The statistical analysis was conducted using SPSS software, version 22. Mean values and 95% confidence intervals (CI) were used to describe continuous data. The Kaplan– Meier method was employed to analyze survival data for each group, with comparison done through the log‐rank and Wilcoxon tests. To compare between the two groups, *t* tests and *p‐*value were utilized. Cox multivariate hazard analysis was utilized to assess the impact of pre‐specified prognostic factors. A significance level of *p‐*value <0.05 was considered. The key raw data have been uploaded to the Research Data Deposit public platform (www.researchdata.org.cn) with approval number RDDA2024932200.

## 3 RESULTS

### 3.1 Clinical features of patients with OTSCC

Between October 9, 2013, and April 26, 2022, the study cohort comprised 56 patients who were collected at Sun Yat‐sen University Cancer Center. Table 1. summarizes patients' clinical characteristics. The study included 41 (73.2%) men and 15 (26.8) women; median age was 50 years (23–79). Most patients were neither smoker (62.5%) nor alcoholics (73.2%). And a lesser percentage of patients have got hypertension (23.2%) and diabetes (5.4%) at the time of diagnosis. Otherwise, tumor was mostly at late clinical T3–T4 tumor stage (76.8%) with clinical lymph nodes (LN) involvement (*n* = 30, 53.6%).

**TABLE 1:** Baseline characteristics (*n* = 56).

Characteristics	No. (%)
Age, years, median (range)	50 (23–79)
≤60	38 (67.9)
>60	18 (32.1)
Sex	
Male	41 (73.2)
Female	15 (26.8)
BMI	
≥24	19 (33.9)
<24	37 (66.1)
Smoking history	
Yes	21 (37.5)
No	35 (62.5)
Alcohol history	
Yes	15 (26.8)
No	41 (73.2)
Hypertension	
Yes	13 (23.2)
No	43 (76.8)
Diabetes	
Yes	3 (5.4)
No	53 (94.6)
Pathological type	
Low to medium differentiated	44 (78.6)
Highly differentiated	12 (21.4)
Tumor location	
Left	33 (58.9)
Right	22 (39.3)
Root	1 (1.8)
cTNM Stage	
II–III	21 (37.5)
IV	35 (62.5)
cT	
1–2	13 (23.2)
3–4	43 (76.8)
cN	
Yes	30 (53.6)
No	26 (46.4)
Family history	
Yes	3 (5.4)
No	53 (94.6)
PNI	
<50.13	15 (26.8)
≥50.13	41 (73.2)
Hypercholesterolemia	
Yes	26 (46.4)
No	30 (53.6)
Hypertriglyceridemia	
Yes	18 (32.1)
dNo	38 (67.9)
Dyslipidemia	
Yes	31 (55.4)
No	25 (44.6)
Metabolic syndrome	
Yes	5 (8.9)
No	51 (91.1)

All patients were assigned to the two groups by Chinese BMI classification.^41^, ^42^ Baseline patients and tumor characteristics between the BMI≥24 and BMI < 24 groups are given in Table 2. At the point of diagnosis, there were 37 patients classified as having a normal weight, while 19 individuals fell into the overweight category. Clinical TNM stage, clinical T stage and LN involvement did not reveal any significant statistical differences between the two BMI groups. Interestingly, we found that overweight patients had significantly higher pCR frequency than normal weight (*p = 0.024*). The same trend was observed that among hypercholesterolemia (*p = 0.003*) and metabolic syndrome (*p = 0.003*) upon initial diagnosis. However, there was no significant difference in prognostic nutrition index (PNI), hypercholesterolemia and dyslipidemia.

**TABLE 2:** Comparison of baseline information between BMI groups.

	*N* (%)		
Characteristics	BMI < 24, *N* = 37, 66.1%	BMI ≥ 24, *N* = 19, 33.9%	*p*
Age (years)			
≤60	22 (59.5)	16 (84.2)	0.06
>60	15 (40.5)	3 (15.8)	
Sex			
Male	28 (75.7)	13 (68.4)	0.562
Female	9 (24.3)	6 (31.6)	
Smoking history			
Yes	14 (37.8)	7 (36.8)	0.942
No	23 (62.2)	12 (63.2)	
Alcohol history			
Yes	12 (32.4)	3 (15.8)	0.183
No	25 (67.6)	16 (84.2)	
Hypertension			
Yes	5 (13.5)	8 (42.1)	0.039
No	32 (86.5)	11 (57.9)	
Diabetes			
Yes	1 (2.7)	2 (10.5)	0.546
No	36 (97.3)	17 (89.5)	
Family history			
Yes	3 (8.1)	0 (0)	0.516
No	34 (91.9)	19 (100)	
Radiotherapy history			
Yes	3 (8.1)	2 (10.5)	1
No	34 (91.9)	17 (89.5)	
Concomitant cancer			
Yes	5 (13.5)	2 (10.5)	1
No	32 (86.5)	17 (89.5)	
Pathological type			
Low to medium differentiated	32 (86.5)	12 (63.2)	0.095
Highly differentiation	5 (13.5)	7 (36.8)	
cTNM stage			
II–III	4 (10.8)	1 (5.3)	0.846
IV	33 (89.2)	18 (94.7)	
cT			
1–2	7 (18.9)	6 (31.6)	0.467
3–4	30 (81.1)	13 (68.4)	
cN			
No	18 (48.6)	8 (42.1)	0.642
Yes	19 (51.4)	11 (57.9)	
pCR			
Yes	10 (27)	11 (57.9)	0.024
No	27 (73)	8 (42.1)	
PNI			
<50.13	12 (32.4)	3 (15.8)	0.183
≥50.13	25 (67.6)	16 (84.2)	
Hypercholesterolemia			
Yes	17 (45.9)	9 (47.4)	0.920
No	20 (54.1)	10 (52.6)	
Hypertriglyceridemia			
Yes	18 (48.6)	15 (78.9)	0.029
No	19 (51.4)	4 (21.1)	
Dyslipidemia			
Yes	18 (48.6)	13 (68.4)	0.159
No	19 (51.4)	6 (31.6)	
Metabolic syndrome			
Yes	0 (0)	5 (26.3)	0.003
No	37 (100)	14 (73.7)	

### 3.2 Survival outcomes of OTSCC patients treated with neoadjuvant anti‐PD1 therapy

**FIGURE 2:**
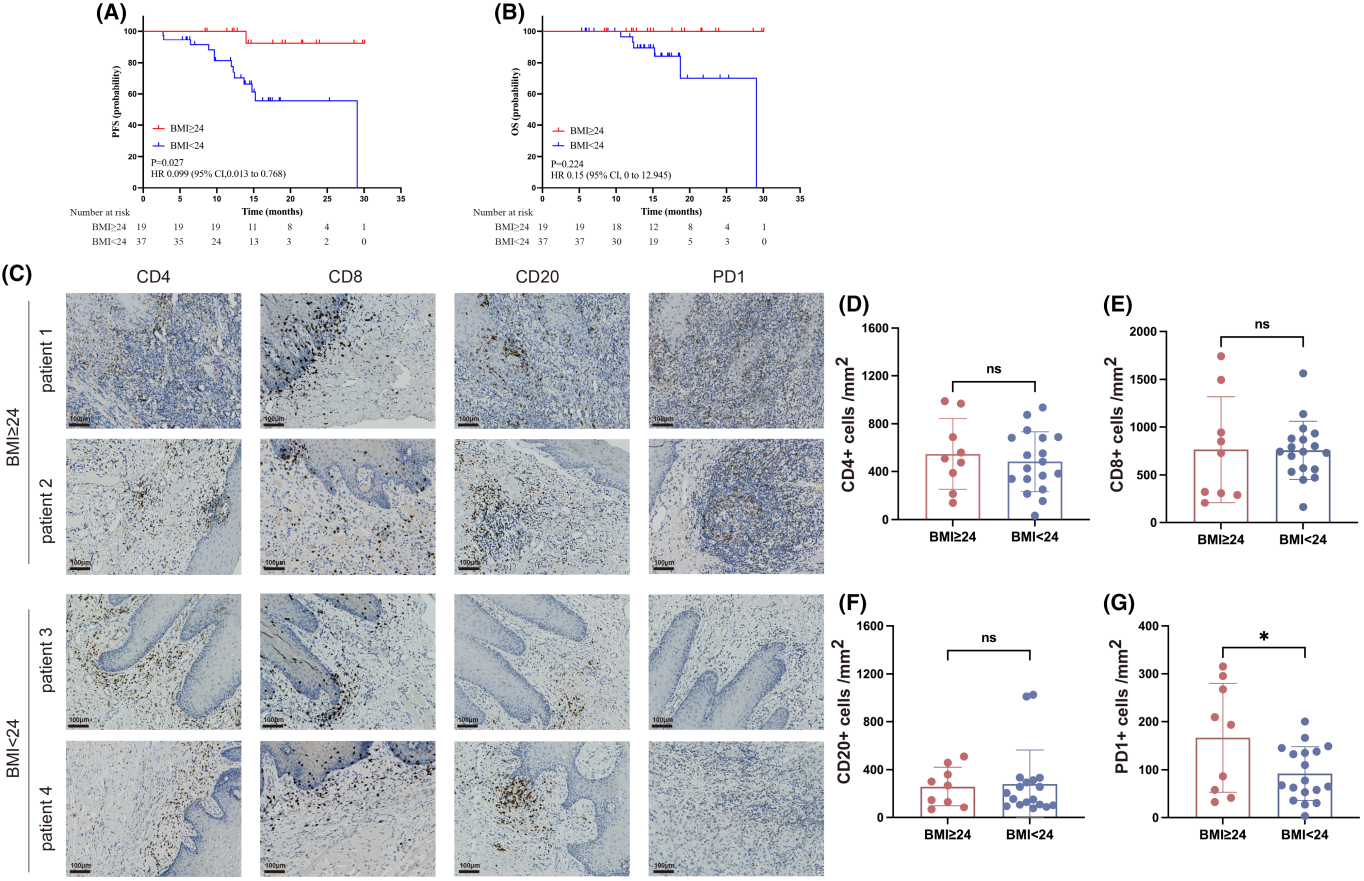
Obese patients benefit from anti‐PD1 therapy by increasing infiltration of PD1+ T cells. (A, B) Kaplan–Meier plots of progression‐free survival (A) and overall survival (B) according to body mass index (BMI) group (BMI < 24 and BMI≥24) in patients with tongue squamous cell carcinoma. (C) Verification of BMI and immune cell infiltration in OTSCC (*n* = 27). immunohistochemical images show the immune cell infiltration (CD4+ T cell, CD8+ T cell, B cell, and PD1+ T cell) in OTSCC tissues. (D–G), human protein quantification analysis results of immunohistochemical staining by Qupath.

The duration until the final follow‐up or mortality varied between 6 and 30 months, with a median duration of 15 months. Among the total of 56 patients, absence of disease was observed in 44 individuals (78.6%) during their most recent follow‐up, 8 (12.5%) patients were alive with disease (five cases recurrence within the local region, two cases of distant metastasis, and one case exhibiting both), 6 (10.7%) died of disease. The findings showed the univariate survival analyses conducted on distinct BMI groups (Figure 2A,B). The effects of BMI on PFS as calculated based on fully adjusted univariate and multivariate Cox regressions (Tables 3, 4). Through univariate analysis, a statistically significant association was observed between high BMI and improved PFS (*p = 0.027*) (Figure 2A; Table 3). Covariates with *p*‐value <0.1 were subjected to multivariable analysis, including BMI, smoking history, radiotherapy history, radiotherapy, cN, alcohol history, hypertension, hypertriglyceridemia and PNI. By multivariate analysis, high BMI remained significantly associated with improved PFS (*p = 0.005*) (Table 4). Besides, we also revealed that smoking history (*p = 0.023*), radiotherapy history (*p = 0.017*) and cN (*p = 0.025*) emerged as independent predictors for PFS (Table 4). But not in other subgroups (Figure S1). Of the whole 56 patients, 6 cancer‐related deaths were all reported in BMI < 24 group, and BMI≥24 group has no death. However, when analyzing the impact of increasing BMI on OS, no statistically significant differences were found (*p* = 0.224) (Figure 2B). These results may be caused by the small‐scale clinical studies and short duration of follow‐up. Hopefully, a large sample size will result in a statistical difference.

**TABLES 3:** Univariate analysis of factors associated with progression‐free survival.

Variables	Progression‐free survival	
	HR (95% CI)	*p*
Sex		
Male	Reference	0.426
Female	1.611 (0.498–5.208)	
BMI		
<24	Reference	0.027
≥24	0.099 (0.013–0.768)	
Age		
≤60 years	Reference	0.868
>60 years	1.097 (0.367–3.278)	
cTNM Stage		
II–III	Reference	0.284
IV	2.010 (0.561–7.210)	
Alcohol history		
No	Reference	0.097
Yes	3.624 (0.791–16.610)	
Smoking history		
No	Reference	0.174
Yes	2.252 (0.699–7.256)	
Radiotherapy history		
No	Reference	0.385
Yes	0.523 (0.113–2.318)	
Hypertension		
No	Reference	0.158
Yes	3.135 (0.641–15.340)	
Diabetes		
No	Reference	0.977
Yes	0.971 (0.126–7.490)	
cT		
1–2	Reference	0.683
3–4	0.785 (0.245–2.509)	
cN		
No	Reference	0.079
Yes	3.147 (0.876–11.310)	
Pathological type		
Low to medium differentiated	Reference	0.167
Highly differentiated	0.030 (0.000–4.341)	
PNI		
Low	Reference	0.206
High	0.503 (0.173–1.459)	
Hypercholesterolemia		
No	Reference	0.938
Yes	0.958 (0.331–2.772)	
Dyslipidemia		
No	Reference	0.247
Yes	0.533 (0.184–1.545)	
Metabolic syndrome		
No	Reference	0.334
Yes	0.038 (0.000–29.130)	

**TABLE 4:** Multivariate analysis of factors associated with progression‐free survival.

Variables	Progression‐free survival	
	HR (95% CI)	*p*
BMI		
<24	Reference	0.005
≥24	0.022 (0.001–0.326)	
Alcohol history		
No	Reference	0.724
Yes	1.469 (0.174–12.442)	
Radiotherapy history		
No	Reference	0.017
Yes	0.030 (0.002–0.530)	
Radiotherapy		
No	Reference	0.056
Yes	5.009 (0.960–26.136)	
cN		
No	Reference	0.025
Yes	13.789 (1.389–136.905)	
Smoking history		
No	Reference	0.023
Yes	14.592 (1.442–147.654)	
Hypertension		
No	Reference	0.386
Yes	3.674 (0.194–69.744)	
Family history		
No	Reference	0.566
Yes	0.481 (0.040–5.838)	
PNI		
Low	Reference	0.273
High	2.761 (0.449–16.964)	

To investigate the effects of BMI on immune infiltration in OTSCC, we measured the expression of CD4, CD8, CD20, PD1 in tumor immune microenvironments (TIMs) involving a total of 27 patients with available of specimen by IHC (Figure 2C). The expression of PD1+ T cells was significantly elevated in high BMI group (Figure 2G), while there were no notable differences detected in the overall expression levels of CD4, CD8 and CD20 among the two groups (Figure 2D–F). These findings demonstrated that obese OTSCC patients may benefit from anti‐PD1 therapy by increasing the infiltration of PD1 + T cells. Obesity is a common cause of chronic inflammation, both systemically and at the tissue level,^43^ and chronic inflammation with increased levels of PD‐1 expression in obese patients may increase the efficacy of anti‐PD1 treatment for OTSCC.

### 3.3 Functional analysis by GSEA and KEGG

**FIGURE 3:**
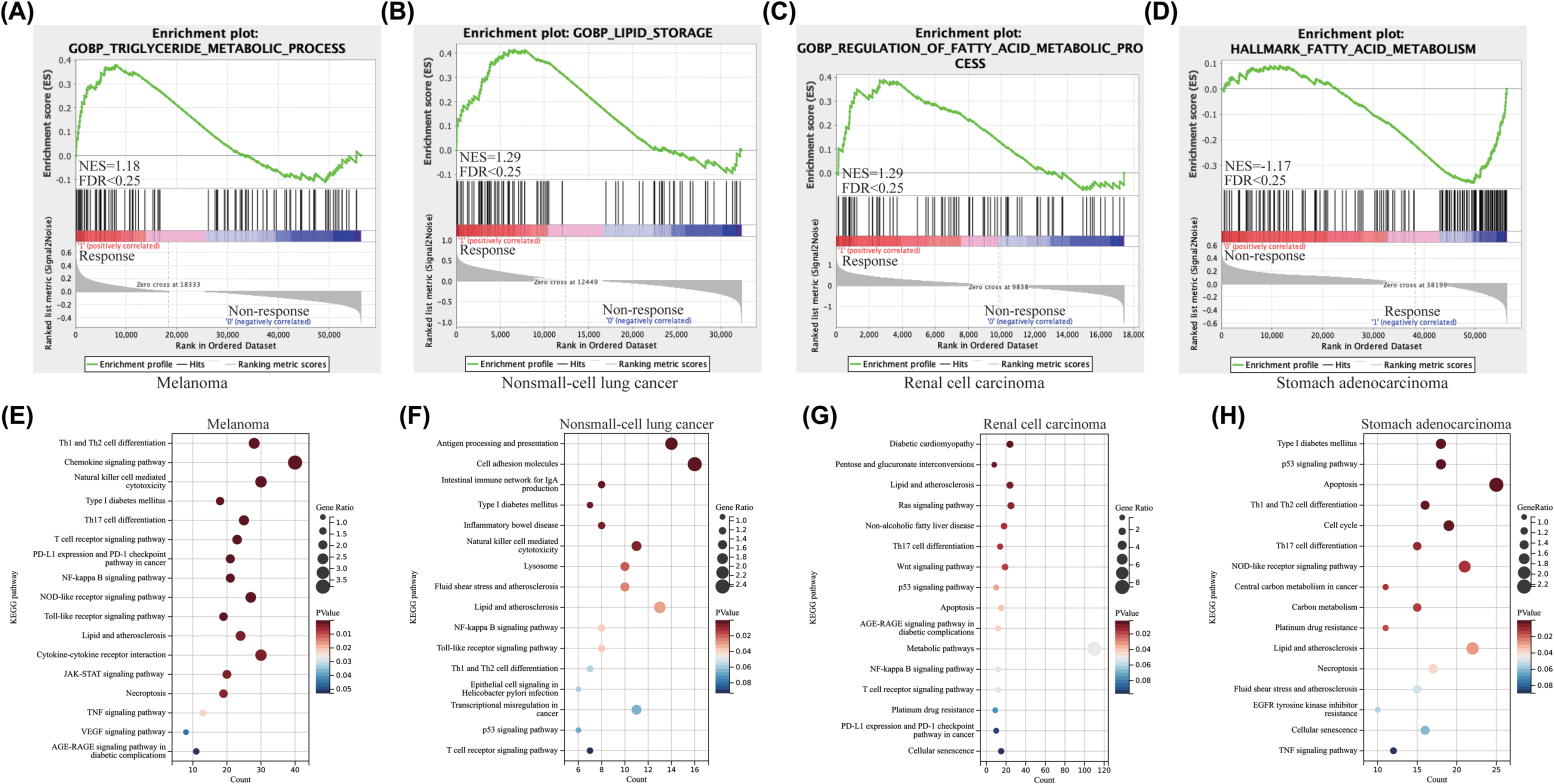
Fatty acid metabolism impacts anti‐PD1 therapy effects in pan‐cancer. (A–D) Result of Gene Set Enrichment Analysis (GSEA) between responders and non‐responder groups in melanoma (A), non‐small cell lung cancer (B), renal cell carcinoma (C) and stomach adenocarcinoma (D). (E–H) Result of Kyoto Encyclopedia of Genes and Genomes (KEGG) between responders and non‐responder groups in melanoma (E), non‐small cell lung cancer (F), renal cell carcinoma (G) and stomach adenocarcinoma (H).

As previous studies described, obesity is defined by an elevated BMI, typically because of excess adipose tissue.^44^, ^45^ And the condition of being obesity leads to a state of meta‐inflammatory characterized by heightened levels of proinflammatory cytokines, glucose, leptin, fatty acids metabolism. These factors have been shown to directly influence the response of T cells.^46^ In this research, we conducted an extensive range of bioinformatics analysis methods to thoroughly investigate the influence of lipid metabolism on the efficacy of anti‐PD‐1 therapy. First, the biological role of lipid metabolism‐associated genes (LMAGs) was illustrated through GSEA in pan‐cancer. We enriched all the pathways associated with lipid metabolism, the findings indicated that among the HALLMARK terms, the responder group exhibited significantly elevated NES values in relation to triglyceride metabolic process, lipid storage, regulation of fatty acid metabolic process and fatty acid metabolism. (Figure 3A–D). Second, we identified DEGs between response and non‐response samples and performed KEGG pathway enrichment analyses. The analysis revealed that the responder group exhibited a significant enrichment in lipid acid metabolism, such as central carbon metabolism in cancer, citrate cycle, insulin resistance, lipid and atherosclerosis, and so on (Figure 3E–H). Collectively, these results illustrate that fatty acid metabolism may play a significant role in anti‐PD1 therapy response in pan‐cancer.

### 3.4 Screening of differentially expressed FAMRGs

**FIGURE 4:**
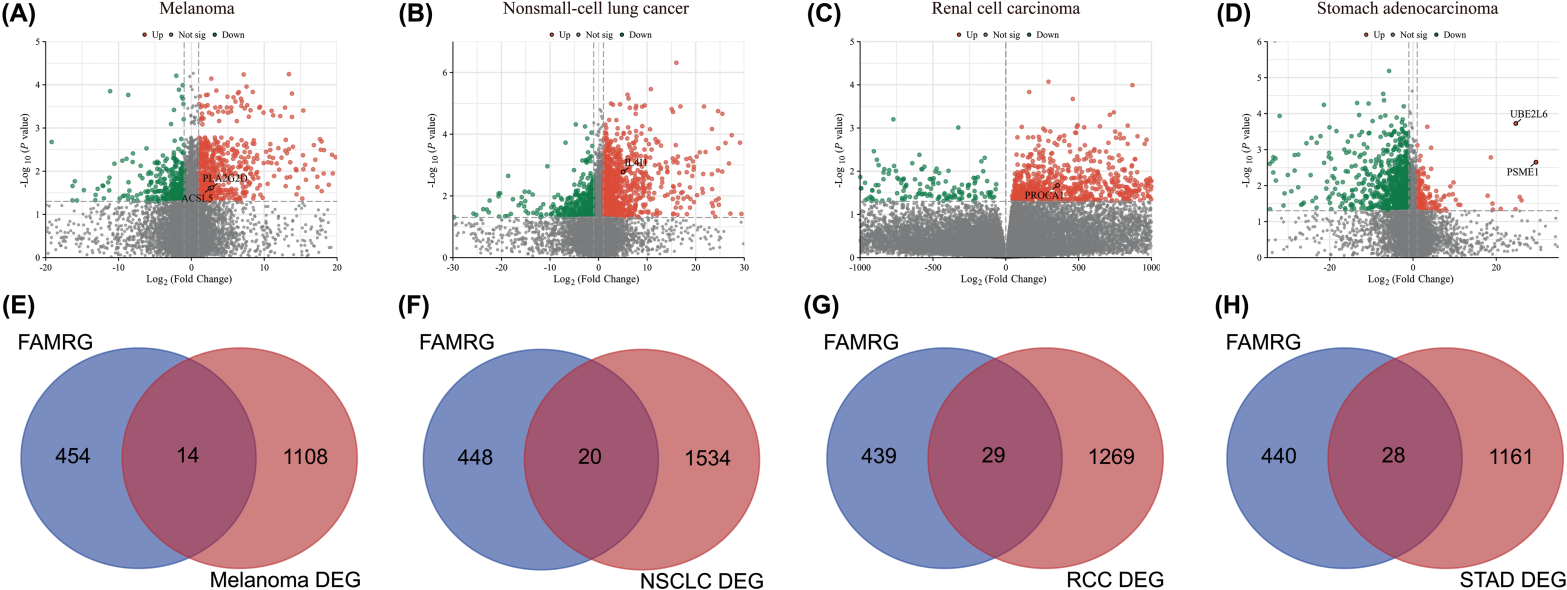
Differentially expressed FAMRGs between responder and non‐responder samples. (A‐D) Volcano map of the differentially expressed genes in pan‐cancer. The red, green, and gray dots indicate high, low, and no difference in expression between responder and non‐responder samples (*p* < 0.05 & |log2 FC|>1). (E–H) Authentication of 91 FAMRGs in the four cancer datasets through Venn diagrams. (Melanoma (A), NSCLC (B), RCC (C), STAD (D)). (The upregulated genes were displayed in red, with downregulated FAMAGs in blue).

To investigate how the fatty acid metabolic process could increase the response rate to immunotherapy. Via limma analysis online tool, we extracted DEGs between responder and non‐responder from PRJEB23709, GSE135222, GSE67501 and PRJEB23709, respectively. The clustering heatmap (Figure S2) and volcano plot depict the differential expression of genes (Figure 4A–D). In total, 468 FAMRGs was downloaded from Molecular Signatures Database (MsigDB) database (Table S1).^40^ Then, Venn diagram software was used to identify the differentially expressed FAMRGs in pan‐cancer datasets, respectively. Overall, there are 91 differentially expressed FAMRGs (Genes that contain duplicates), including 14 FAMRGs in melanoma samples, 20 FAMRGs in NSCLC samples, 29 FAMRGs in RCC samples and 28 FAMRGs in STAD samples (Figure 4E,F; Table 5).

**TABLE 5:** All 91 differentially expressed genes (DEGs) of FAMRGs were detected from 4 cancer datasets.

Cancer type	Genes Name
Melanoma	MAPKAPK2 MAOA UBE2L6 KMT5A IL4I1 ACSL5 PSME1 PLA2G2D SYK NUDT19 PTPRG PON2 TWIST1 SUCLA2
NSCLC	GAPDHS GK MAPKAPK2 BDH2 UBE2L6 IL4I1 GABARAPL1 ACSL5 PHYH PSME1 PLA2G2D SYK PTPRG ACSF3 ABCC1 HSDL2 P2RX7 TWIST1 PNPLA2 CBR1
RCC	SLCO2B1 PON1 PLIN2 SLCO3A1 HSD17B4 PPARG CBR4 SLC43A3 PECR MTLN ACOT9 ECI2 ELOVL4 IVD PROCA1 GCDH SLC22A5 PPT2CR ABP2 CYP4F22 PTGR1 CRYZ HADHB ACSF3 AIG1 AKR1C3 MECR BCKDHB CBR1
STAD	ANXA1 MDH1 PLA2G2A TPI1 PTGES3 SCD5 UBE2L6 GOT2 DLST MDH2 PHYH RDH11 PSME1 METAP1 GGT1 ERP29 HACD3 PLA2G15 ACAT2 IDI1 PTPRG CPOX AKT1 ECHDC2 HADHA CPT1A LTA4H RAP1GDS1

### 3.5 Identification of hub FAMRGs associated with the effectiveness of anti‐PD1 treatment

**FIGURE 5:**
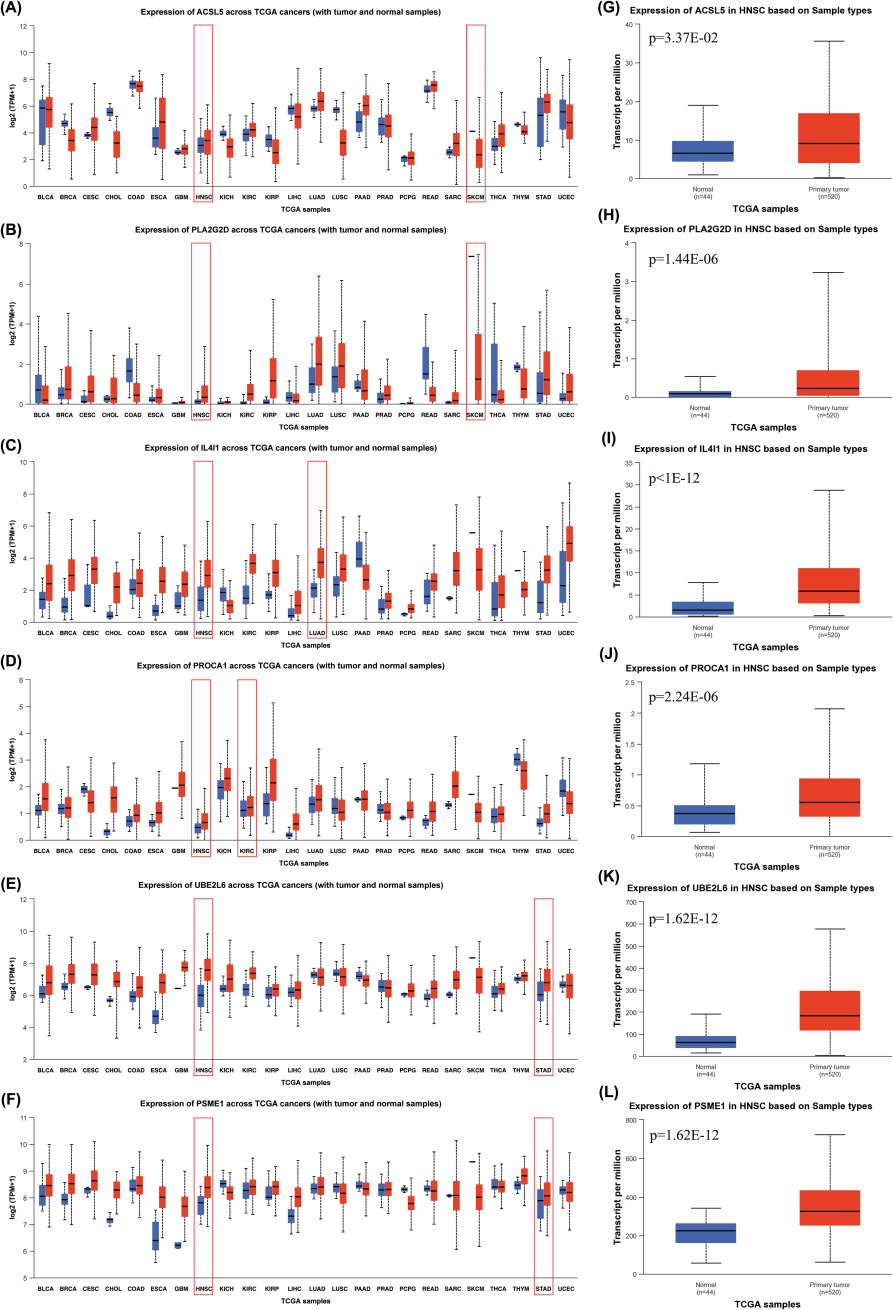
(A–F) Histogram of hub FAMRGs expression in 24 types of unpaired normal and normal tissues from TCGA using Wilcoxon rank‐sum test. (G–L) Histograms of hub FAMRGs in normal and HNSCC with significant differences from UALCAN portal.

**FIGURE 6:**
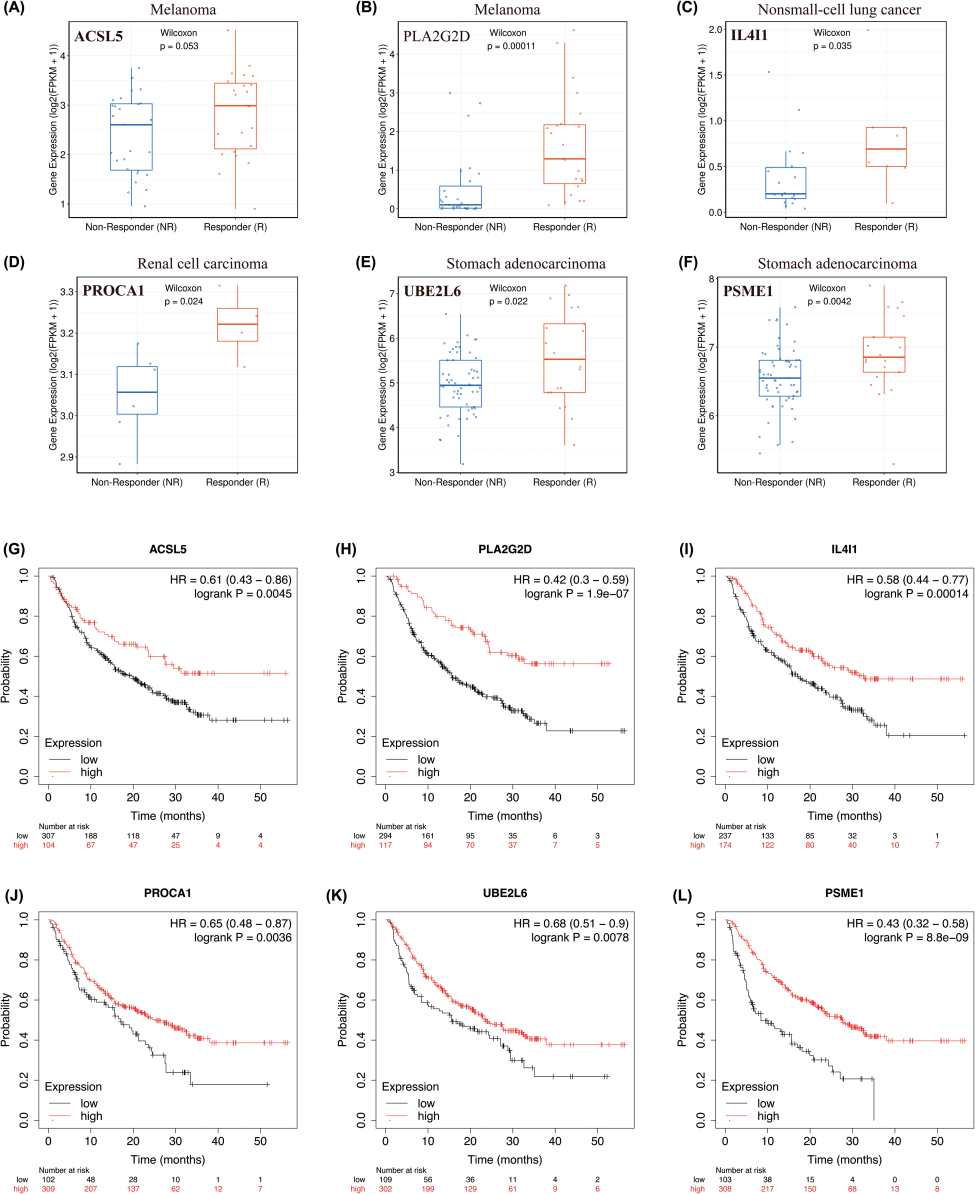
Correlation of hub FAMRGs expression with anti‐PD1 therapy response and prognostic value in cancer samples. (A, B). ACSL5 and PLA2G2D expression levels between responder and non‐responder samples in melanoma. (C) IL4I1 expression levels between responder and non‐responder samples in non‐small cell cancer. (D) PROCA1 expression level between responder and non‐responder samples in renal cell carcinoma. (E, F) UBE2L6 and PSME1 expression level between responder and non‐responder samples in stomach adenocarcinoma. (G–L) The Kaplan–Meier curves of the low and high FAMRGs expression in pan‐cancer patients treated with anti‐PD1 therapy (*n* = 520).

A total of 91 FAMRGs with differential expression were identified based on the Venn diagrams. To ascertain the correlation between FAMRGs and the therapeutic efficacy of PD‐1. First, we explored the functions of these genes in fatty acid metabolism (Table S2), second, we compare FAMRGs expression in human adjacent normal versus 24 types of tumor tissues by UALCAN database, the result showed that ACSL5, PLA2G2D, IL4I1, PROCA1, UBE2L6 and PSME1 were significantly upregulated in the most cancer types from TCGA (Figure 5A–F), especially in HNSCC sample (Figure 5G–L). Third, we used TIGER database (http://tiger.canceromics.org/) to compare differentially expressed FAMRGs. These results further showed that higher ACSL5 and PLA2G2D expression in melanoma responder samples compared to that in non‐responder samples. Meanwhile, the same result showed that the expression of IL4I1, PROCA1, UBE2L6 and PSME1 expression were notably elevated in responder tissues compared to non‐responder tissues in NSCLC, RCC and STAD, respectively (Figure 6A–F). Additionally, the overall survival analysis was conducted by Kaplan–Meier plotter database (http://kmplot.com/analysis/index.php?p=background), the results demonstrated that ACSL5 high expression in patients treated with anti‐PD1 therapy significantly prolonged OS compared to low expression, the same trend were also found in PLA2G2D, IL4I1, PROCA1, UBE2L6 and PSME1 (Figure 6G–L). Finally, these 6 FAMRGs were included in the biomarkers in predicting prognosis the efficiency of anti‐PD1 therapy.

**FIGURE 7:**
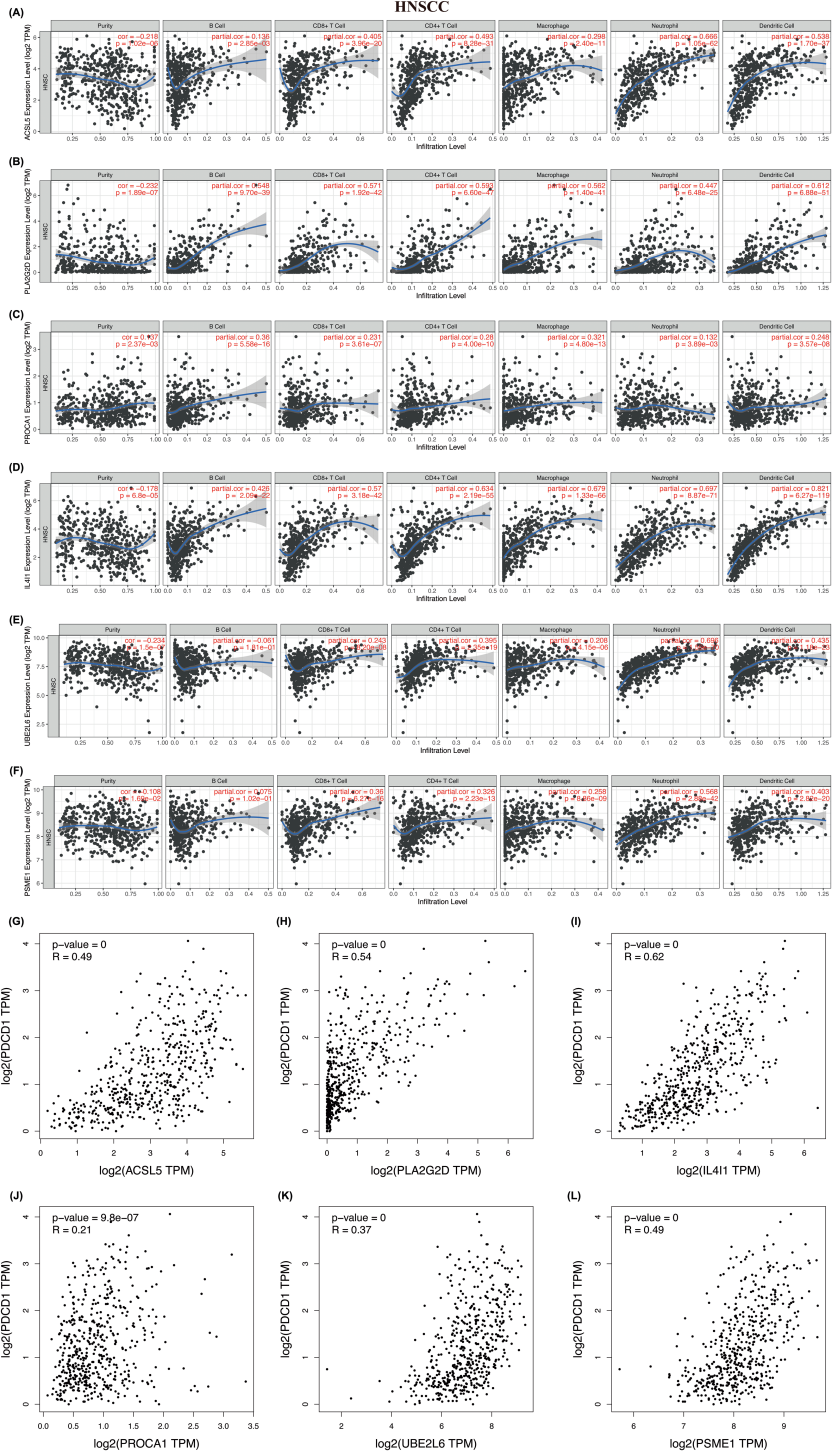
FAMRGs affect anti‐PD1 therapy efficiency through T cells infiltration. Correlation analysis of hub FAMRGs (A, ACSL5, B, PLA2G2D, C, IL4I1, D, PROCA1, E, UBE2L6, F, PSME1) and 7 immune cells infiltration from TIMER in HNSCC samples, including B cells, CD8+ T cells, CD4+ T cells, macrophages, neutrophils, dendritic cells. (G–L), Pearson correlation analysis between hub FAMRGs and PD‐1 level in HNSCC Patient characteristics.

To explore whether the FAMRGs affect immune cell infiltration in tumor microenvironment, we conducted an analysis using the TIMER database to investigate the infiltration of 6 distinct immune cell types, including B cells, CD8+ T cells, CD4+ T cells, macrophages, neutrophils, and dendritic cells. The findings indicated a positive association between the expression of ACSL5 expression and CD8+ T cell (*r* = 0.405, *p* = 3.96e‐20), CD4+ T cell (*r* = 0.493, *p* = 8.28e‐31), negativity correlated with tumor purity (Figure 7A). Meanwhile, we evaluated the other hub FAMRGs with these 7 immune cells. The results demonstrated that the levels of PLA2G2D, PROCA1, IL4I1, UBE2L6 and PSME1 exhibited a positive correlation with CD8+ T cell, CD4+ T cell (Figure 7B–F).

To illustrate whether FAMRGs is correlated with the T cell exhaustion biomarker PD1 in HNSCC. We used GEPIA database to evaluate correlation of FAMRGs and PD1 in HNSCC samples. The result showed that ACSL5, PLA2G2D, PROCA1, IL4I1, UBE2L6 and PSME1 (Figure 7G–L) were positively correlated with PD1, respectively. In summary, the above results suggested that fatty acid metabolic process might affect the anti‐PD1 therapy efficiency through regulating immune cell infiltration, especially T cell exhaustion.

## 4 DISCUSSION

In the present study, we reveal that BMI levels can independently serve as indicators for predicting the efficacy of anti‐PD1 therapy in patients with OTSCC. Consistently, we confirm that BMI is positively correlation with PD1+ T cell in tumor microenvironment (TME). In mechanism, the GSEA and KEGG analyses revealed enrichment of fatty acid metabolism pathways in the responder group. Furthermore, we identified 6 FAMRGs (ACSL5, PLA2G2D, IL4I1, PROCA1, UBE2L6, PSME1) which were high expressed in anti‐PD1 therapy responders, presented a positive correlation with PD1 expression and the infiltration of immune cell. We believe that the results of this study provide the first dataset that assesses and validates the prognostic significance of BMI in predicting responses to immunotherapies in tongue cancer. Furthermore, our study highlights the critical role of fatty acid metabolism and T cell exhaustion in shaping the efficacy of immunotherapies.

Obesity is currently defined by an elevated BMI, typically because of excess adipose tissue (AT). In current research, ATs are characterized as heterogeneous organs that have a significant impact on the regulation of metabolism,^47^ inflammation,^48^ and anti‐tumor immune responses.^49^ But, in recent years, some experts have identified an “obesity paradox”, wherein there is a simultaneous increase in the prevalence of obesity‐associated malignancies and studies investigating the impact of obesity on the efficacy of available antineoplastic therapies.^50^ The response to immune checkpoint blockade (ICB) is generally more favorable among patients with obesity‐associated BMI compared to lean patients in NSCLC, melanoma and renal cell carcinoma.^51^ This interesting research also reported in head and neck cancer that obesity is an important risk factor,^52^ but cancer immunotherapy may exhibit greater anti‐tumor efficacy in obese patients.^53^, ^54^, ^55^ Recent evidence suggested BMI at the time of clinical diagnosis was found to be an independent predictive factor for recurrence/metastasis head and neck squamous cell carcinoma patients using pembrolizumab, and the patients with normal weight have better prognosis than underweight.^56^ Our findings are in accord with recent studies indicating that the response rate for immunotherapy in OTSCC patients with high BMI was much higher than patients with low BMI. The most importantly, to date, we found for the first time the prognostic contribution of BMI in OTSCC with immunotherapy.

In addition, we also explore the underlying mechanisms. As described above, by both clinical samples and online datasets analyses, our study showed that an increased prevalence of PD1+ exhausted CD8 T cells in the obese patients have better response to anti‐PD1 therapy. There are similarities with the current study, some researchers found that obesity‐associated increases in systemic leptin were responsible for promoting CD8 T cell exhaustion, as evidenced by elevated surface expression of PD‐1 on CD8 tumor‐infiltrating lymphocytes and loss of cytokine secretion and cytolytic activity.^28^ While other studies have suggested that the chronically inflamed obese state and subsequent generation of exhausted T cells may enhance tumor progression while concurrently promoting an environment conductive to ICIs. Additionally, exhausted T cells can be subdivided into multiple populations based on their re‐activation potential, and some subpopulations are more responsive to PD‐1 blockade than others.^57^, ^58^ In summary, although some of these phenotypes may underlie ICI efficacy in obese cancer patients, the proposition that obesity‐mediated T cell exhaustion in reversed by checkpoint blockade is likely an over‐simplification. Therefore, a large number of studies is needed to further explore the mysteries and allow more patients to benefits from immunotherapy.

As is known to all, obesity is an abnormality of systematic metabolism. Obesity has been proved to induce increased levels of leptin, which affects anti‐tumor immunity by increasing PD‐1 expression and promoting T cell exhaustion.^28^ While these impacts immune disorder, they allow for strengthened restoration of T cell activity following anti‐PD1 therapy.^59^ It is a prove that systemic metabolism influences the tumor microenvironment (TME) and impact anti‐tumor immunity. Moreover, recent research has demonstrated that CD8+ effector T cells oxidize more fatty acids by the leptin STAT3 axis, which suppresses anti‐tumor immune responses in breast cancer.^60^ Thus, the aim of this research was to investigate the influence of obesity on the response to anti‐PD1 therapy. This study did not find any notable variances in the infiltration of CD8+ T cells between two distinct groups when stratified by BMI, however, we show that increased PD‐1+ T cells have been associated with the responsiveness to anti‐PD1 therapy in the OTSCC clinical data. It is also possible that heightened PD‐1 expression on tumor‐infiltrating lymphocytes form patients with obesity simply provides an increased number of targets for engagement of anti‐PD1 antibodies.^28^ Furthermore, we found local tumor metabolism influences the efficacy of anti‐PD1 therapy, and we obtain 6 FAMRGs positively associated with PD1 expression in OTSCC. At present, our study suggested that genes associated with fatty acid metabolism have the potential to serve as biomarkers for predicting and evaluating the effectiveness of immunotherapy in OSTCC.

According to the FAM molecular subtypes, we have identified 6 FAMRGs (ACLS5, PLA2G2D, PROCA1, IL4I1, UBE2L6) that could significantly contribute to the response of HNSCC patients undergoing anti‐PD1 therapy. Among the 6 hub FAMRGs, Prior research has indicated that the protein product produced by ACSL5 gene is responsible for converting unbound long chain Fatty Acids into fatty acyl‐coenzyme A, and plays a role in both the uptake of Fatty Acid and the synthesis of triacylglycerol.^61^ ACSL5 involved in lipid metabolism and suggesting that it may play a vital role.^62^, ^63^, ^64^ Besides, it has been reported by studies that ASCL5 has been identified as a potential prognostic factor and predictor of response to immunotherapy in cases of pancreatic and cutaneous melanoma cancer.^37^, ^65^ Analogously, our current findings in OTSCC align with previous studies indicating a positive correlation between the expression of PLA2G2D and immune infiltration, as well as a better prognosis observed in HNSCC, breast cancer, and cervical squamous cell carcinoma,^66^, ^67^, ^68^ which aligns with the outcomes we have currently observed in OTSCC. Moreover, previous research revealed that the lipid metabolism‐associated prognostic signature has potential as a reliable biomarker for forecasting the effectiveness of chemotherapy and anti‐PD‐L1 therapy in colorectal carcinoma, which includes PROCA1.^69^ The effects of IL4I1, UBE2L6 and PSME1 on immunotherapy have rarely been reported. We discovered these three newly potential predictor genes that may control the immunotherapy efficacy. Importantly, our research unveiled that these 6 hub FAMRGs possess the potential to serve as biomarkers for prognosticating the efficacy of immunotherapy in patients with HNSCC.

Although our findings are unprecedented, certain limitations are worth mentioning. Firstly, the limited number of clinical studies on neoadjuvant anti‐PD1 therapy in OTSCC patients is primarily attributed to the delayed incorporation of immunotherapy into their treatment protocol. In the future, we aim to recruit more patients who have undergone this treatment for further validation. Secondly, this study lacks the relevant molecular mechanism, and we will explore it in the future. Thirdly, although BMI has been widely used as a surrogate for obesity, it does not reflect more specific measures and the distribution of adipose tissue. Obesity can be more precisely defined by measuring body fat percentage or by medical imaging to assess the fat content in the future. We will delve deeper into this question in further research.

## 5 CONCLUSION

We identified that BMI could serve as promising prognostic biomarkers in OTSCC patients undergoing immunotherapy. And our findings demonstrate that obesity have profound effects on efficacy of anti‐PD1 therapy by regulating PD1+ T cell infiltration. In addition, OTSCC patients with enhancing fatty acid synthesis metabolism were more likely to respond to anti‐PD1 therapy by high expression of 6 FAMRGs, including ACSL5, PLA2G2D, IL4I1, PROCA1, UBE2L6, and PSME1. Thus, our study provides some novel and efficient biomarkers in predicting prognosis and in the efficiency of anti‐PD1 therapy, thus guiding to an effective therapeutic strategy and facilitating personalized immunotherapy in the future.

## AUTHOR CONTRIBUTIONS

**Xiyan Tan:** Data curation (equal); formal analysis (equal); investigation (equal); methodology (equal); validation (equal); visualization (equal); writing – original draft (lead); writing – review and editing (equal). **Guoli Li:** Data curation (equal); formal analysis (equal); investigation (equal); methodology (equal); validation (equal); visualization (equal); writing – review and editing (equal). **Honghao Deng:** Investigation (equal). **Guoming Xiao:** Investigation (equal). **Yaqin Wang:** Funding acquisition (equal); writing – review and editing (equal). **Chenzhi Zhang:** Conceptualization (equal); data curation (equal); funding acquisition (equal); methodology (equal); project administration (equal); resources (equal); supervision (equal); validation (equal); writing – review and editing (equal). **Yanfeng Chen:** Conceptualization (equal); data curation (equal); funding acquisition (equal); methodology (equal); project administration (equal); resources (equal); supervision (equal); validation (equal); writing – review and editing (equal).

## FUNDING INFORMATION

This work was supported by the National Natural Science Foundation of China (grant numbers 82,203,003), the Natural Science Foundation of Guangdong Province (grant numbers 2021A1515010602) and the Health & Medical Collaborative Innovation Project of Guangzhou City (202201011261).

## CONFLICT OF INTEREST STATEMENT

The authors declare no conflict of interest.

## ETHICS STATEMENT

The study was approved by the Sun Yat‐sen University Cancer Center Institutional Review Board and in accordance with the Helsinki declaration.

## Supporting information


**Figure S1.** Kaplan–Meier plotter of PFS in different subgroups.
**Figure S2.** The heatmap of the top 50 DEGs between responder and non‐responder samples across four cancer types.

**Table S1.** Catalog of genes associated with fatty acid metabolism from MsigDB.

**Table S2.** Functional synopsis of differentially expressed FAMRGs.

## Data Availability

All the data are available in a public, open‐access repository.
