# Supplementary material for: Obesity enhances the response to neoadjuvant anti‐PD1 therapy in oral tongue squamous cell carcinoma
Source: Cancer Med. 2024 Jun 24;13(12):e7346. doi: 10.1002/cam4.7346 (PMC11194614; doi:10.1002/cam4.7346)
Supplement: Supplementary file 1 — Figure S1. Kaplan–Meier plotter of PFS in different subgroups. Figure S2. The heatmap of the top 50 DEGs between responder and non‐responder samples across four cancer types. [file CAM4-13-e7346-s002.docx]

**
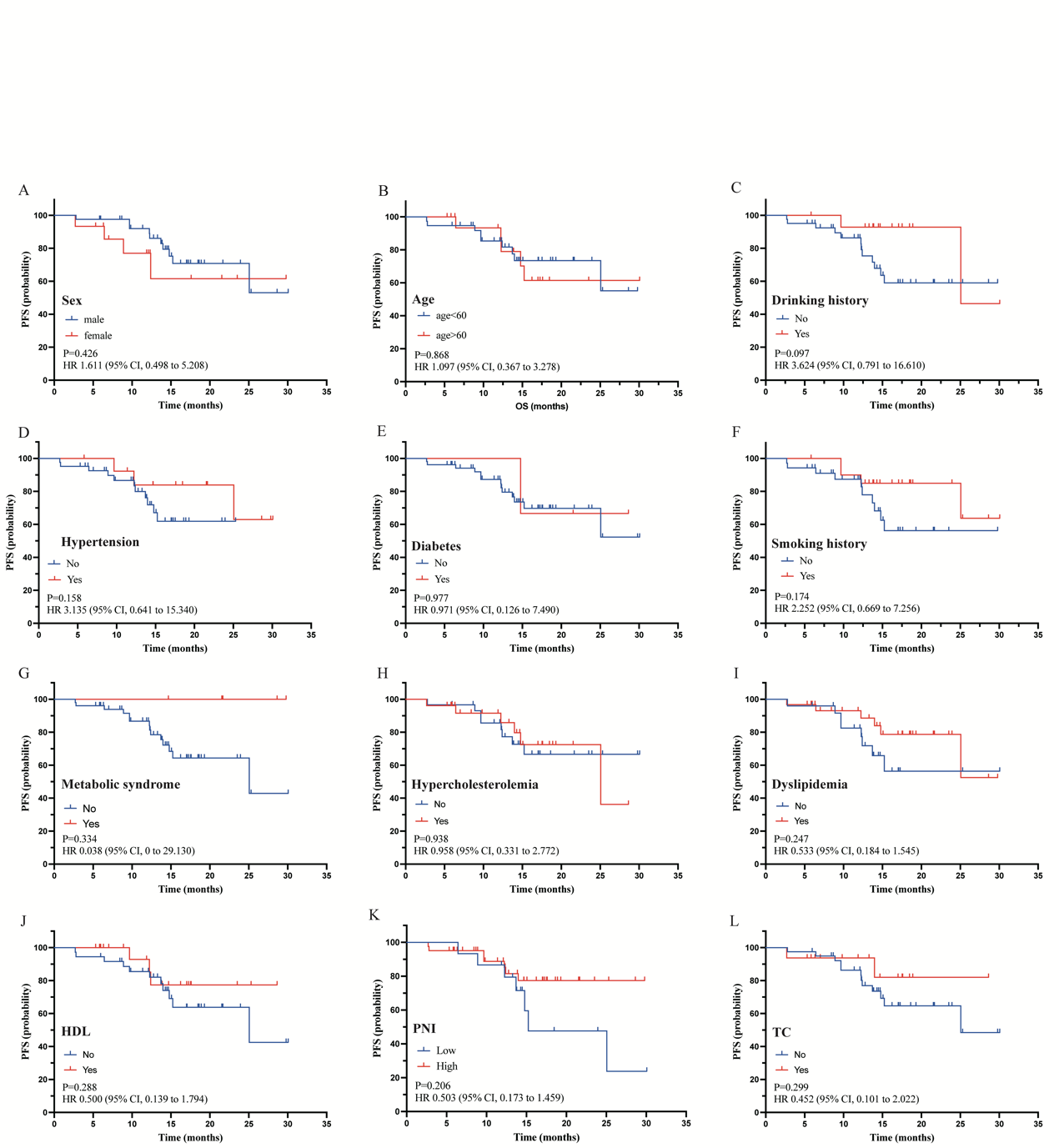
****Supplementary Figure 1. Kaplan-Meier plotter of progression-free survival according to sex (A), age (B), drinking history (C), hypertension (D), diabetes (E), smoking history (F), metabolic syndrome (G), hypercholesterolemia (H), dyslipidemia (I), HDL (J), PNI (K), TC (L).**

**
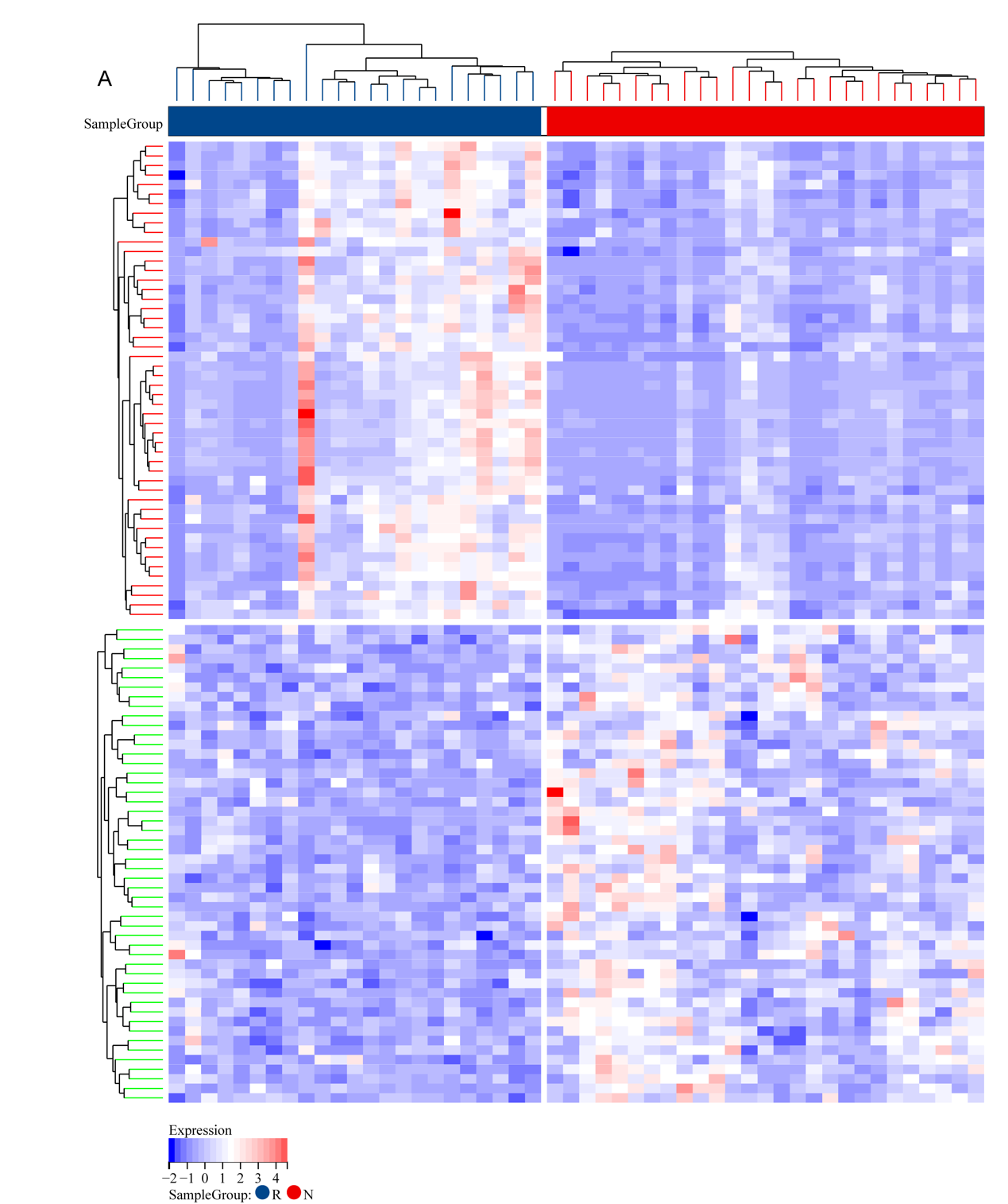
**

**
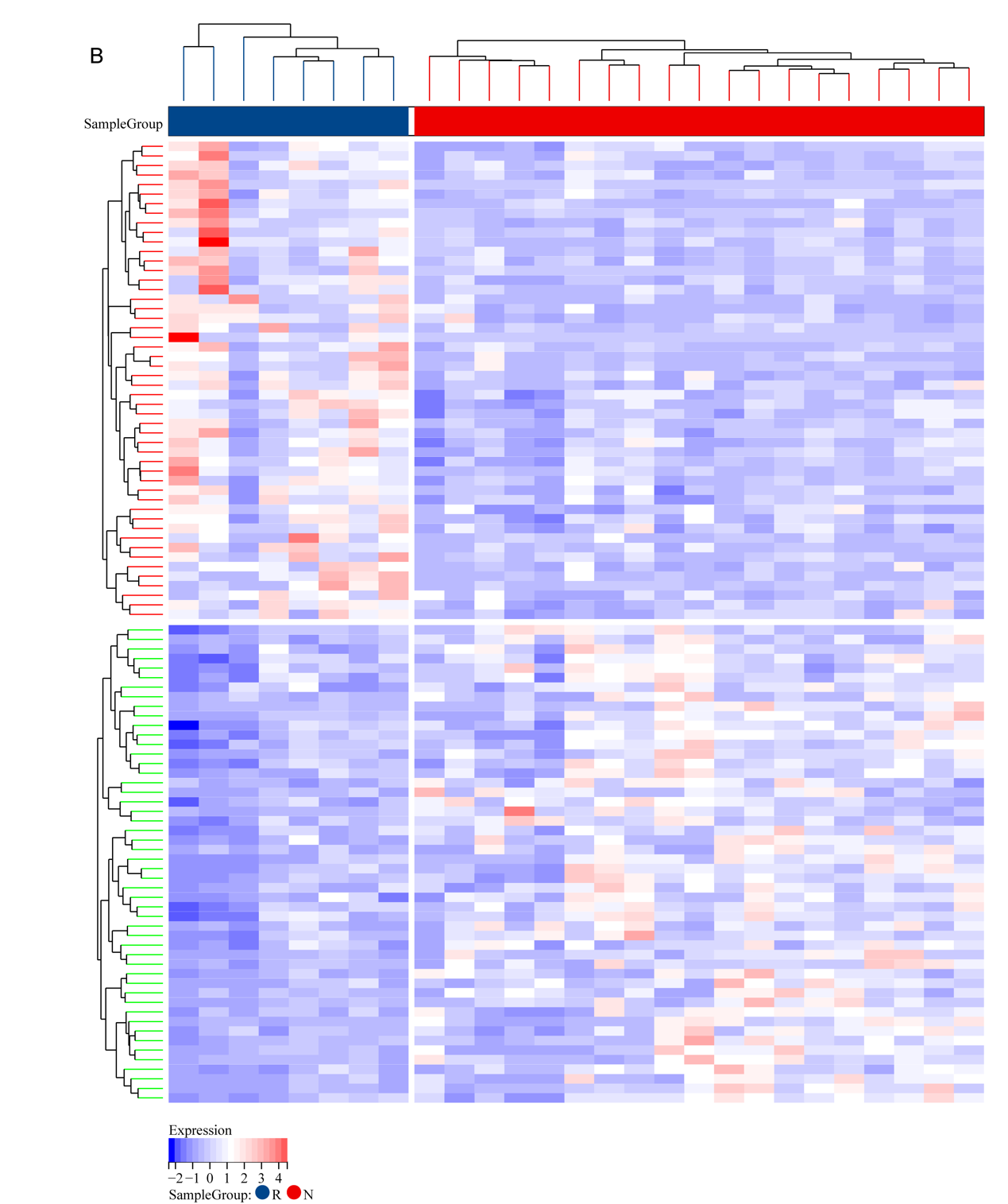
**

**
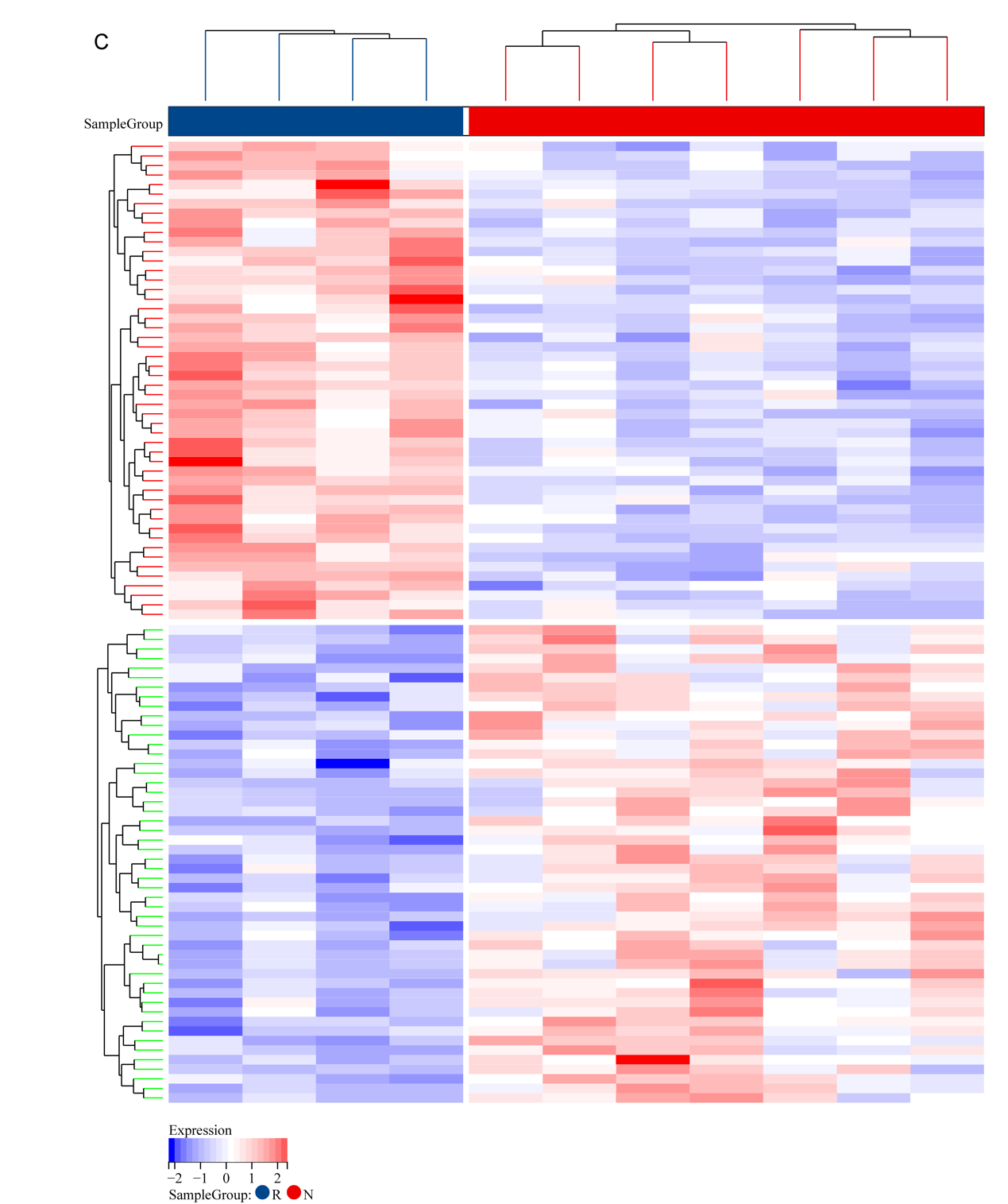
**

**
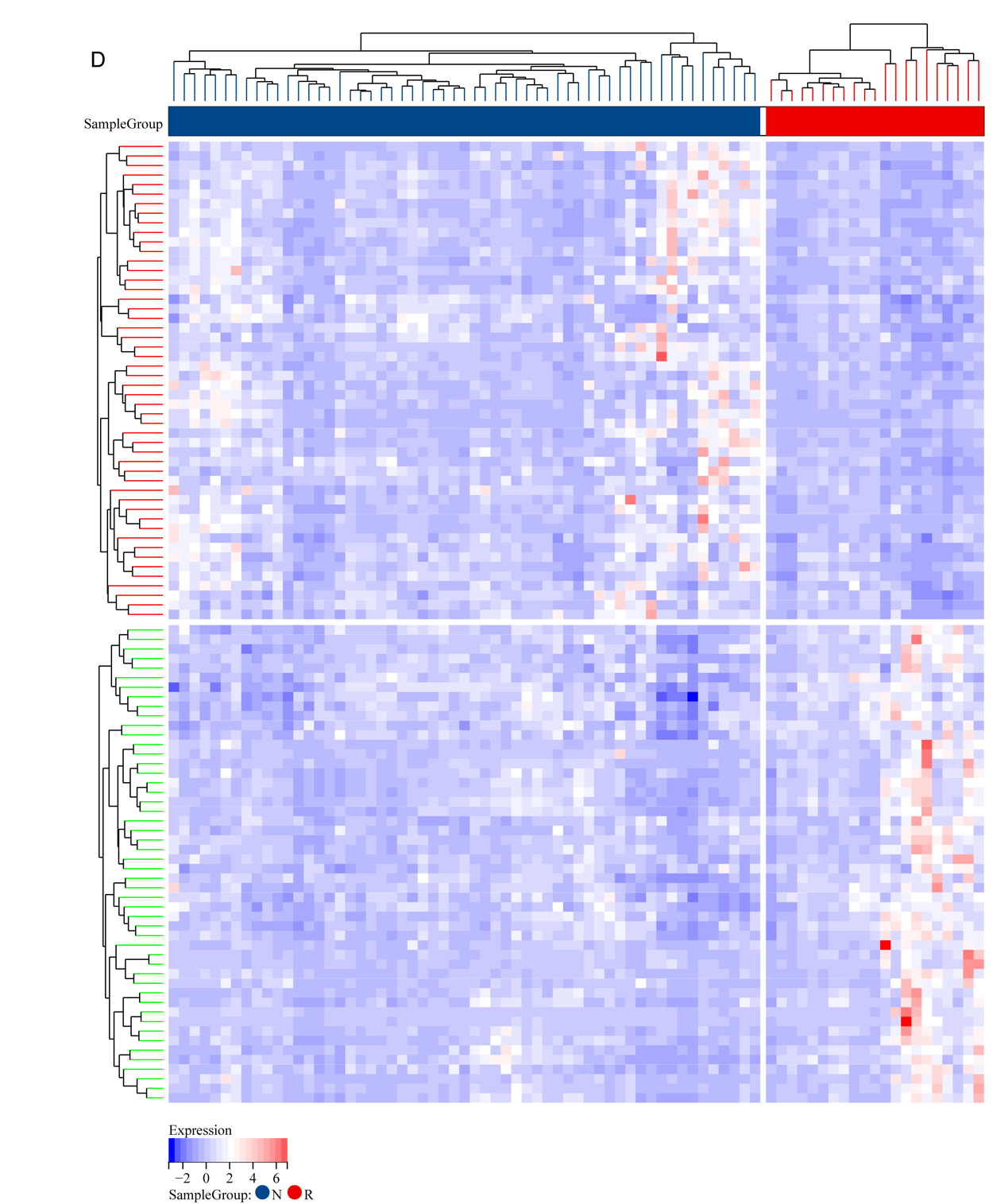
**

**Supplementary Figure 2. The heatmap of the top 50 DEGs between responder and non-responder samples in melanoma (A), non-small cell lung cancer (B), renal cell carcinoma (C) and stomach adenocarcinoma (D).**
